# The Genetic Code: Francis Crick’s Legacy and Beyond

**DOI:** 10.3390/life6030036

**Published:** 2016-08-25

**Authors:** Koji Tamura

**Affiliations:** 1 Department of Biological Science and Technology, Tokyo University of Science, 6-3-1 Niijuku, Katsushika-ku, Tokyo 125-8585, Japan; koji@rs.tus.ac.jp; Tel.: +81-3-5876-1472; 2 Research Institute for Science and Technology, Tokyo University of Science, 2641 Yamazaki, Noda, Chiba 278-8510, Japan

Francis Crick (Figure 1) was born on 8 June 1916, in Northampton, England, and passed away on 28 July 2004, in La Jolla, California, USA. This year, 2016, marks the 100th anniversary of his birth. A drastic change in the life sciences was brought about by the discovery of the double helical structure of DNA by James Watson and Francis Crick in 1953 [1], eventually leading to the deciphering of the genetic code [2]. The elucidation of the genetic code was one of the greatest discoveries of the 20th century. The genetic code is an algorithm that connects 64 RNA triplets to 20 amino acids, and functions as the Rosetta stone of molecular biology.

At the age of 60, Crick moved to La Jolla from Cambridge, England, and shifted his focus to the brain and human consciousness. He tackled this subject for the last 28 years of his life. His life-long interest was the distinction between the living and the non-living, which motivated his research career. Crick was arguably one of the 20th century’s most influential scientists, and he devoted himself to science until his death.

Francis Crick continued to exercise his intellectual abilities throughout his life. His research style was characterized by collaborations with outstanding partners, James Watson in discovering the structure of DNA, Sydney Brenner in cracking the genetic code, Leslie Orgel in probing the origins of life, and Christof Koch in understanding human consciousness. Francis Crick was never modest in his choice of scientific problems [3] and was like “the conductor of the scientific orchestra” [4]. He always discussed his ideas, which helped in the progress he made in science. Interestingly, his son, Michael, then 12 years old, was the first person to read the earliest written description of the genetic code. Crick wrote the following in a letter to Michael,

“…*Now we believe that the D.N.A. is a code. That is, the order of the bases (the letters) makes one gene different from another gene (just as one page of print is different from another). You can now see how Nature makes copies of the genes. Because if the two chains unwind into two separate chains, and if each chain then makes another chain come together on it, then because A always goes with T, and G with C, we shall get two copies where*…”

(Figure 2).

This is the fundamental principle of biology. The big questions that arose after the discovery of the structure of DNA were “how is the code used?” and “what is it a code for?” Francis Crick turned his attention to find answers to these questions for the next 13 years. George Gamow, who is famous for the Big Bang theory, founded the 20-member “RNA Tie Club” with Watson, to discuss the transmission of information by DNA. RNA-illustrated neckties were provided to all members, and a golden tiepin with the abbreviation for one of the 20 amino acids was given to each member. Crick was “TYR” (tyrosine). Crick’s famous “adaptor hypothesis” was prepared for circulation in the RNA Tie Club [5], but when Paul Zamecnik and collaborators discovered transfer RNA (tRNA) [6], Crick did not believe that it was indeed the adaptor, because of its unexpectedly large size. Crick insisted that there would be 20 different adaptors for the amino acids, and that they would bring the amino acids to join the sequence of a nascent protein. A manuscript entitled “Ideas on protein synthesis (October, 1956)” remains extant (Figure 3). Crick spoke about “The Central Dogma” at a Society for Experimental Biology symposium on “The Biological Replication of Macromolecules”, held at the University College London in September, 1957. The Central Dogma holds true even today, and is another example of Crick’s genius.

In 1961, Francis Crick, Sydney Brenner, Leslie Barnett, and Richard Watts-Tobin first demonstrated the three bases of DNA code for one amino acid [7]. That was the moment that scientists cracked the code of life. However, ironically, the first decoding of the “word” of the genetic code was reported in the same year by a non-member of the RNA Tie Club, Marshall Nirenberg, who spoke at the International Biochemical Congress in Moscow. Matthew Meselson heard Nirenberg’s 15-minute talk in a small room and told Crick about it. Crick arranged for Nirenberg to give the talk again at the end of the meeting. Starting with Nirenberg and Heinrich Matthaei’s work [8], followed by that of Nirenberg and Philip Leder [9], the decoding was completed by Har Gobind Khorana [10]. Finally, Brenner, Barnett, Eugene Katz, and Crick placed the last piece of the jigsaw puzzle of life by proving that UGA was a third stop codon [11].

Thus, the genetic code was cracked, and it is the greatest legacy left behind by Francis Crick, along with the discovery of the double helical nature of DNA. As hallmarks of the foundation of molecular biology, they will continue to shine forever. However, the origin and evolution of the genetic code remain a mystery, despite numerous theories and attempts to understand them. In the mid-1960s, Carl Woese proposed the “stereochemical hypothesis”, which suggested that the genetic code is derived from a type of codon–amino acid pairing interaction [12]. On the other hand, Crick proposed the “frozen accident hypothesis” and conjectured that the genetic code evolved from the last universal common ancestor and was frozen once established. However, he explicitly left room for stereochemical interactions between amino acids and their coding nucleotides, stating that “It is therefore essential to pursue the stereochemical theory…vague models of such interactions are of little use. What is wanted is direct experimental proof that these interactions take place…and some idea of their specificity” [13].

What is the real origin of the genetic code? tRNAs and aminoacyl-tRNA synthetases play fundamental roles in translating the genetic code in the present biological system [14], but what could have been the primitive forms of these molecules? Although Crick thought that tRNA seemed to be nature’s attempt to make RNA do the job of a protein [2], the primordial genetic code prior to the establishment of the universal genetic code might have resided in a primitive form of tRNA. Such an example of “operational RNA code” [15] may be seen as a remnant in the acceptor stem of tRNA, which still functions as a critical recognition site by an aminoacyl-tRNA synthetase [16,17,18]. In addition, why are 20 amino acids involved in the genetic code? Discrimination of an amino acid with the high fidelity attained by modern aminoacyl-tRNA synthetases (error rate as low as 1/40,000 [19]) would be impossible using a simple thermodynamic process alone, because the hydrophobic binding energy of a methylene group is, at the most, ~1 kcal/mol. Therefore, several sets of amino acids with similar side chains might have been coded non-selectively in the primitive stage [20]. Furthermore, the genetic code is the relationship between left-handed amino acids and right-handed nucleic acids. As non-enzymatic tRNA aminoacylation has been shown to occur chiral-selectively [21], the establishment of the genetic code might be closely associated with the evolutionary transition from the putative “RNA world” to the “RNA/protein world” in terms of homochirality [22]. All these are critical issues that should be investigated in the future. 

The life force of Francis Crick was once described as similar to the “*incandescence* of an intellectual nuclear reactor” [23]. His passion for science is an inspiration for future scientific explorers. The Guest Editor of this Special Issue dedicates all articles included herein to the memory of Francis Crick.

## Figures and Tables

**Figure 1:**
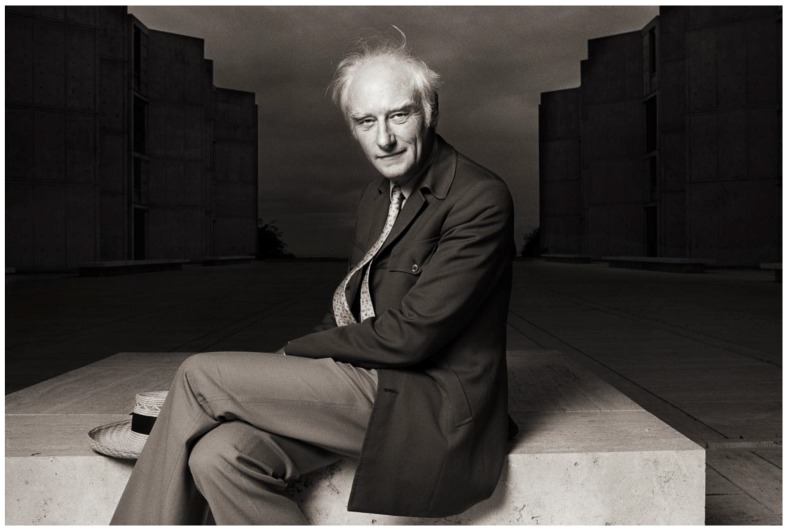
Sir Francis Crick, La Jolla 1982, Photograph by Norman Seeff. Credit: Norman Seeff Productions.

**Figure 2:**
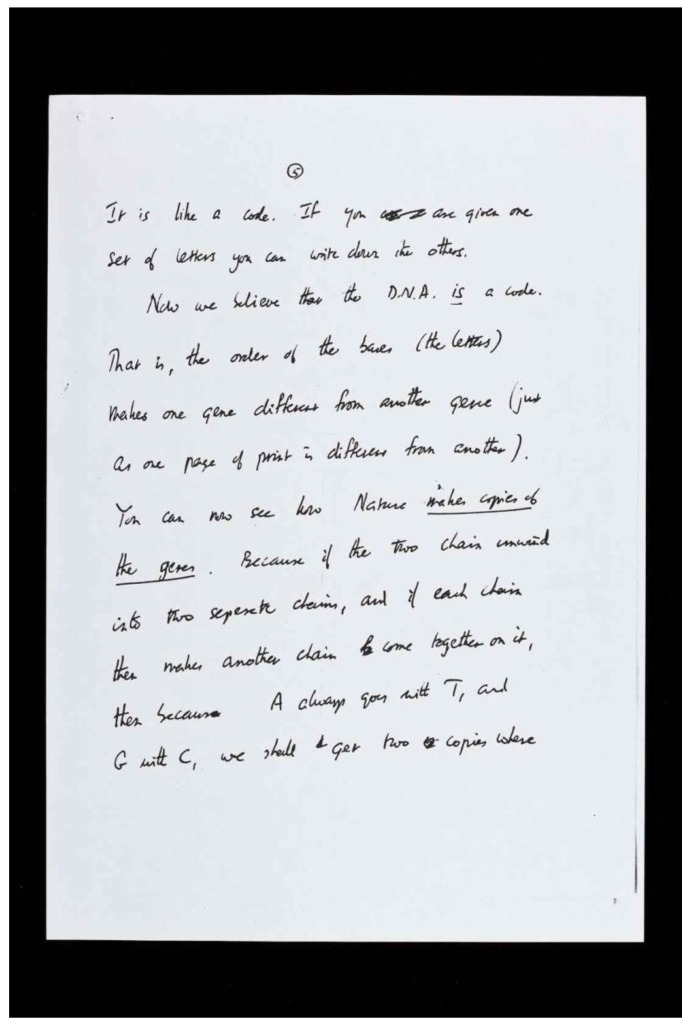
Letter from Francis Crick to his son, Michael, explaining his and Watson’s discovery of the structure of DNA. The letter is the earliest written description of the genetic mechanism on 19 March 1953. Credit: Wellcome Library, London.

**Figure 3:**
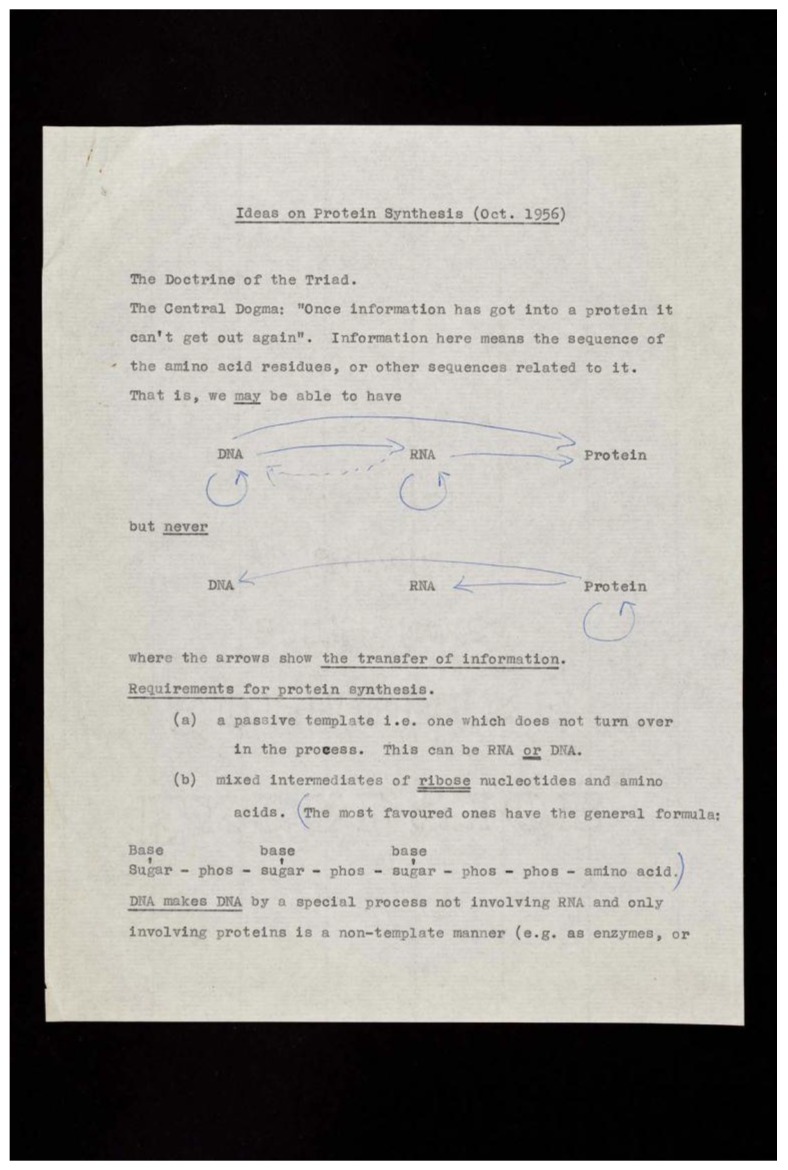
The earliest written description of “The Central Dogma” in a manuscript entitled “Ideas on protein synthesis (October 1956)”. Credit: Wellcome Library, London.
